# Trends in research approaches and gender in plant ecology dissertations over four decades

**DOI:** 10.1002/ece3.11554

**Published:** 2024-06-11

**Authors:** Urmi Poddar, Kristi Lam, Jessica Gurevitch

**Affiliations:** ^1^ Department of Ecology and Evolution Stony Brook University Stony Brook New York USA; ^2^ Roslyn High School Roslyn Heights New York USA; ^3^ Department of Forestry and Natural Resources Purdue University West Lafayette Indiana USA

**Keywords:** big data, gender ratio, meta‐analysis, meta‐research, plant ecology research trends, research methods, systematic review

## Abstract

Dissertations are a foundational scientific product; they are the formative product that early‐career scientists create and share original knowledge. The methodological approaches used in dissertations vary with the research field. In plant ecology, these approaches include observations, experiments (field or controlled environment), literature reviews, theoretical approaches, or analyses of existing data (including “big data”). Recently, concerns have been raised about the rise of “big data” studies and the loss of observational and field‐based studies in ecology, but such trends have not been formally quantified. Therefore, we examined how the emphasis on each of these categories has changed over time and whether male and female authors differ in the methods employed. We found remarkable temporal consistency, with observational studies being dominant over the entire time span examined. There was an increase in the number of approaches employed per dissertation, with increases in analyses of databases and theoretical studies adding to rather than replacing traditional methodologies (like observations and field experiments). The representation of women increased over time. There were some differences in the approaches taken by men and women, which requires further investigation.

## Graphical abstract

Recently, some researchers have raised concerns over the apparent rise of big data studies and the decline of field observations in ecology. But to what extent is this change occurring? Using plant ecology dissertations as a test case, we quantified trends in research methodologies and found that observational studies continue to dominate, even though newer methods have nudged their way in.

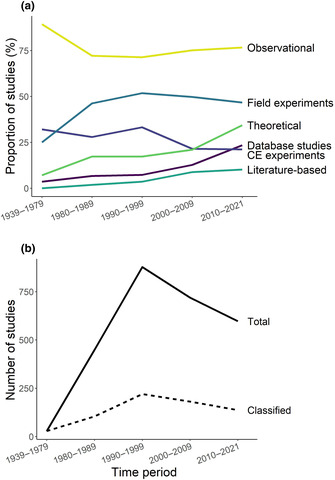

## 1 INTRODUCTION

### 1.1 The nature of data

Science relies on both theory and data. Data provide the information that guides, informs, and tests theory and understanding. The source of the data on which the science of ecology relies has expanded from emphasizing individual‐investigator observational studies and controlled‐environment experiments, to single‐investigator field experiments, and recently, to multi‐investigator studies and the analysis of “Big Data.” Scientific evidence also depends on the synthesis and interpretation of existing literature. Traditional literature reviews, systematic reviews, and meta‐analyses contribute to these efforts. Focusing on plant ecology, we ask, how has the nature of scientific evidence changed over time? If evidence is the basis for our understanding, the very nature of the evidence must change the way we inform theory, student training, textbook content, and our conceptualization of the nature of our field. What are the ways in which data are accumulated and analyzed in graduate student training? The graduate experience is the foundation for any discipline, and graduate student research is indicative of how we put evidence together to resolve problems and advance understanding. In addition to the inherent interest in understanding the course of research approaches in this discipline, we believe that knowing where data—the basis for scientific evidence—comes from and how it is produced offers important insights into the scientific process more generally.

Concern over whether analysis of large, online datasets (“Big Data”) has supplanted experimental and field work in ecological graduate training and, more broadly, in ecological research has gained attention in recent years. Some authors have argued for a greater emphasis on big data to accelerate progress in ecology (e.g., Farley et al., 2018; Hampton et al., 2013), while others have urged for more caution (e.g., Lewis et al., 2018; Mouquet et al., 2015; Robertson & Feick, 2018), with the fear that basic knowledge is being lost. But to what extent has this shift in emphasis actually occurred?

What other changes have arisen in ecological research? For example, how much does personal observational work, the backbone of historical work in plant ecology, remain a part of graduate training? Direct observational and field studies are particularly important for documenting patterns in today's changing natural world, and the loss of such studies may hinder our ability to understand and respond to anthropogenic changes and conservation threats. Recent studies have quantified trends in research topics in ecology and related fields (e.g., Anderson et al., 2021; Carmel et al., 2013; Kim et al., 2018; McCallen et al., 2019; Nobis & Wohlgemuth, 2004; Westgate et al., 2020), but to the best of our knowledge, trends in research methodologies employed have received much less attention (but see Geldmann et al., 2020; Ríos‐Saldaña et al., 2018; Spiegelberger et al., 2012).

The extent of the participation of women in ecological research has also been documented in recent years (e.g., Farr, 2017; Lupon et al., 2021; Salerno et al., 2019), but studies on the graduate training and research of men and women are lacking. We believe that it is important to document whether male and female doctoral students use different research methods, as this may indicate differences in the graduate training they receive and/or in their research interests. Moreover, such differences may also have implications for their future careers. For example, women are underrepresented in computation‐heavy fields such as computer science (National Science Board, NSF, 2022), and therefore, it is possible that they are also underrepresented among the users of computational and theoretical methods within plant ecology. If that is the case, then female doctoral students in this field may miss out on learning computational skills, which are an important transferrable skill in today's world (Yadav et al., 2017).

### 1.2 Plant ecology as a test case: Have the data we use to inform science changed?

Using plant ecology as a test case, we studied the changes in the methods used in dissertations to evaluate how the focus of graduate work has changed over time and how the nature of the data and evidence produced has changed.

We focused on plant ecology for several reasons. This field has a long history in ecology, and many of the ecological core concepts were originated by plant ecologists (e.g., Clements, 1916; Tansley, 1935, 1947). The broader field of ecology was too large and too general for this project. Two of us (UP and JG) work in the area of plant ecology, and one of us (JG) has observed the changes in gender and approaches in this discipline over many decades (e.g., Gurevitch, 1988). We examined dissertations for several reasons. Dissertations are a foundational scientific product. They indicate the areas in which early‐career scientists are being trained and in which they are producing original knowledge. They are inherently single‐authored, so the attribution of the work and the approaches taken are unambiguous. They are far less subject to publication bias than is published literature. Dissertations typically contain more than one chapter, each of which may have been undertaken using different methodological approaches, allowing consideration of the mix of methodologies used at different time periods. They proffer an indication of where a field of science has been and suggest where it is headed. To our knowledge, they have not been searched systematically for methodologies in ecological research prior to the present study.

To address our questions, we carried out a systematic review of doctoral dissertations in plant ecology from 1939, the earliest record available, through 2021. The availability of dissertations written before 1980 was sparse, hence most of our analysis focused on the past 40 years by necessity. We also restricted our study to English language dissertations. After sampling and selecting appropriate dissertations, we categorized all of the methodological components of each dissertation. We further wanted to understand how the participation of and approaches taken by men and women might differ. Hence, in addition, we also determined whether dissertation authors were male or female and which approaches were taken by male and female authors.

### 1.3 Trade‐offs in generality, realism, and precision

Levins (1966) discussed the tradeoffs in generality, realism, and precision in scientific models, saying, “It is of course desirable to work with manageable models which maximize generality, realism, and precision toward the…goals of understanding, predicting, and modifying nature. But this cannot be done” (Levins, 1966). Consequently, he advocated sacrificing one of the three so that the other two could be emphasized in developing a model. We add a fourth: sometimes models may be valued for heuristic value, even if generality, realism, and precision are sacrificed. For example, exponential population increase and Lotka‐Volterra models primarily have heuristic value, and while familiar to every ecologist, are certainly neither realistic nor general nor precise. We propose that Levins' categorization of models also applies to the *data* we use to understand, predict, and manage nature. What we learn from data also trade‐off in realism, generality, and precision and is sometimes most valued for its heuristic use.

How do different kinds of data provide conceptually different information on which we inform our understanding of ecology? Experiments have long been considered the gold standard for resolving scientific questions. They are the most rigorous way to test hypotheses and disentangle potential confounding factors. Controlled‐environment experiments provide precision with control of most factors but may have limited generality and realism (e.g., see Voelkl et al., 2020). On the other hand, field experiments have the advantage of realism, but face other issues (Hurlbert, 1984), including limited precision, sample size, and spatial scales. Typically, only the manipulated variable is controlled, with all else being uncontrolled. Some of these limitations can be overcome by coordinated experiments at multiple sites by multiple researchers. Such networks of researchers are an important new approach to combining the advantages of experimental work with the ability to generalize across geography and ecological systems (Fraser et al., 2013).

Studies based on direct, unmanipulated observations (even if quantitative) are the basis for documenting patterns and hypothesizing processes, with approaches ranging from natural history to complex quantitative analyses. Such observational data have always been a mainstay in plant ecology and are particularly important in situations where manipulative experiments cannot be carried out. While observational studies have high realism, they may not be able to disentangle confounding factors.

What we refer to as “big data” are studies based on the increasing number of large databases for ecological and other data. The accumulation and availability of these databases have enabled important broad‐scale analyses and generalizations. However, these data are often essentially observational. They are rarely the result of manipulative experiments in which hypotheses are tested and confounding and correlated factors are separated experimentally. Kendall (2015) observed that ecologists are increasingly inferring causality from observational data, that “big data” are typically observational rather than experimental, and that observed variables may covary and be confounded for reasons other than causality. Soranno and Schimel (2014) discuss the increasing use of “big data” in ecology and the compromises inherent in different strategies for studying large, complicated systems.

Theoretical studies, including mathematical modeling and simulations, can vary in their generality, precision, and realism. Such studies provide the raw material for generating new hypotheses and can test scenarios that cannot be directly observed or experimented upon. Marquet et al. (2014) propose that efficient theories can advance environmental science in the era of big data, and in a follow‐up commentary, they (Marquet et al., 2015) express the concern that there is a “mounting dismissal of the value of theory in the biological sciences… this is particularly acute in systems biology and ecology.” However, whether this dismissal is in fact occurring, and if so, whether it is reflected by a marked decline in the development of theory in Ph.D. dissertations, is not known.

## 2 METHODS

### 2.1 Dissertations in ecology as a window into changing methodological approaches

The *Proquest Dissertations & Theses Global Database* (https://www.proquest.com/pqdtglobal; ProQuest LLC, 2022) was used to find relevant dissertations. The website was accessed on January 4, 2022, and the following search string was used:

((SU(plant? OR vegetation OR tree OR leaf OR botan* OR flora* OR seedling* OR grass*) OR TI(plant? OR vegetation OR tree OR leaf OR botan* OR flora* OR seedling* OR grass*)) AND (SU(ecology OR ecolog* OR ecosystem? OR communit* OR conservation OR diversity OR biodiversity OR range OR trait?) OR TI(ecology OR ecolog* OR ecosystem? OR communit* OR conservation OR diversity OR biodiversity OR range OR trait?)) AND LA(en OR eng OR english)) NOT (“water reclamation” plant? OR “water treatment” plant? OR econom* OR bioengineer* OR biotechnolog* OR “bacterial flora”).

The search results were further filtered as follows:

*Manuscript type*: Doctoral dissertations; *Language*: English.

*Subject*: NOT (plant pathology AND genetics AND agronomy AND microbiology AND soil sciences AND zoology AND plant propagation AND molecular biology AND business community AND livestock AND wildlife management AND animal behavior AND physical geography AND cartography AND fish production AND behavioral sciences AND civil engineering AND enzymes).

This search returned 5423 results. These dissertations were then manually screened for metadata completeness (e.g., author name, year of publication) and availability of abstracts. Dissertations with incomplete metadata, particularly those missing an abstract or year of publication, or those that were not in English, were removed. This left us with 3832 studies. These were then screened for relevance, based on whether the study focused on a topic in plant ecology. This was done by reading titles and abstracts. Relevant studies were defined as those that focused on one or more plant species or communities and that studied the interactions of those plant species/communities with other organisms or with the environment. Only studies on embryophytes (i.e., bryophytes and vascular plants) were included. Other taxonomic groups that have been traditionally included under the term “plants,” such as algae and fungi, were excluded. After screening for relevance, 2670 dissertations remained.

Methods are not discoverable using key words and other conventional search approaches but require careful reading. Hence, we could only classify a limited number of dissertations. Therefore, we started by selecting 20% of the relevant dissertations at random from each decade after 1980 (plus all studies before 1980, as there were very few of them). After classifying this initial set of studies, an additional 5% were randomly chosen and classified; as the proportions among categories were stable at that point (Table S2), we felt that this was a sufficient sample of the approaches taken for the total population of studies for each decade. In total, 670 dissertations were classified by methodology.

Each selected dissertation was classified into the following categories: (1) observational, (2) field experiment, (3) controlled‐environment experiment, (4) literature‐based, (5) database analysis (including “big data”), and (6) theoretical (see Table 1 for definitions). Note that a single dissertation could fall into multiple categories. Classification was carried out primarily by reading abstracts. However, if the abstract did not contain sufficient information for unambiguous classification, relevant parts of the full text were used, if available. If a thesis had both insufficient information in the abstract and a lack of full‐text availability, it was removed and replaced by another randomly chosen dissertation from the same decade.

**TABLE 1:** Definitions of terms.

Category	Definition
Observational	Studies in which researchers observed a natural phenomenon or studied a dependent variable of interest without manipulating the independent variable/s of interest
Field experiment	Experiment carried out in a natural environment. An experiment is a study in which independent variable/s of interest are manipulated, and the effects of such manipulations on dependent variable/s of interest are observed Outdoor experiments in man‐made/partially man‐made settings (e.g., gardens, agricultural fields) were also considered field experiments, as they are subject to factors beyond the researchers' control (such as weather conditions)
Controlled‐environment experiment	Experiments in controlled environments, generally indoors. For example, growth chamber and greenhouse experiments
Database analysis/“Big data”	Studies in which the researchers carried out statistical analyses on data collected by previous studies. This usually involved compiling data from different sources/studies, or using one or more databases
Literature‐based (Literature review/systematic review/meta‐analysis)	Studies in which the researchers synthesized the results of previous studies on a given topic or research question (in addition to the introductory literature review that is usually required for most dissertations). This could be either done qualitatively (narrative review) or quantitatively (systematic review and meta‐analysis) The difference between the previous category and this one is that the former uses raw data from previous studies while this one involves synthesizing and summarizing the results of previous studies
Theoretical (mathematical modeling/simulations)	Studies that used mathematical and statistical modeling and/or simulations

The classified dissertations were divided into 10‐year time periods based on the year of publication. Then, the number and percentage of dissertations in each category for each time period were calculated. These numbers were used to assess the prevalence of different scientific approaches in the overall dataset and over time. We also calculated the number of methodological approaches used per study, as a measure of the “diversity” of methodologies used in a single dissertation. Studies from 2020 to 2021 were placed in the 2010s time period. Studies from before 1980 were grouped into a single period due to the small number of such studies.

### 2.2 Did gender ratio in plant ecology change over time and with methodology?

The gender classification, in contrast to the methodological classification, was carried out on the entire dataset of all 2760 relevant dissertations. The gender of each thesis author was determined using genderize.io (https://genderize.io/; Demografix ApS, 2019), a web application that uses social media records to predict gender from given names. This program has been used in other recent studies on gender disparities in scientific research (e.g., Cevik et al., 2021; Holman et al., 2018; Huang et al., 2020) and it has a minimum accuracy of around 80% (Karimi et al., 2016; Sebo, 2021). Along with a gender classification, the program also provides the probability of the name belonging to that gender and the number of past records of that name in the application's database. To reduce the chances of false classification, we set a probability cut‐off of 0.8 and a past record cut‐off of 50 (names that fell below either of these cutoffs were considered undermined). For authors whose gender could not be determined by their first names, we re‐ran the algorithm on their middle names (if available). Where gender could not be determined from either first name or middle name, we looked up online profiles (e.g., institutional profile, lab website, etc.) of the author and attempted to determine gender from their photographs. Any remaining undetermined records were removed from this analysis.

Using this data, we determined the gender ratio of plant ecology Ph.D. authors and looked at how the ratio of men to women has changed over time. A Chi‐squared goodness of fit test was used to determine whether these gender ratios significantly differed from a 1:1 ratio (equal male and female representation). We also considered whether different methodologies were more or less likely to be used by either gender, using the dissertations that had been classified by methodology. This was done by comparing the gender ratio of authors in each methodology category to the overall gender ratio using Chi‐squared goodness of fit tests.

## 3 RESULTS

### 3.1 Observational studies continue to dominate in plant ecology

**FIGURE 1:**
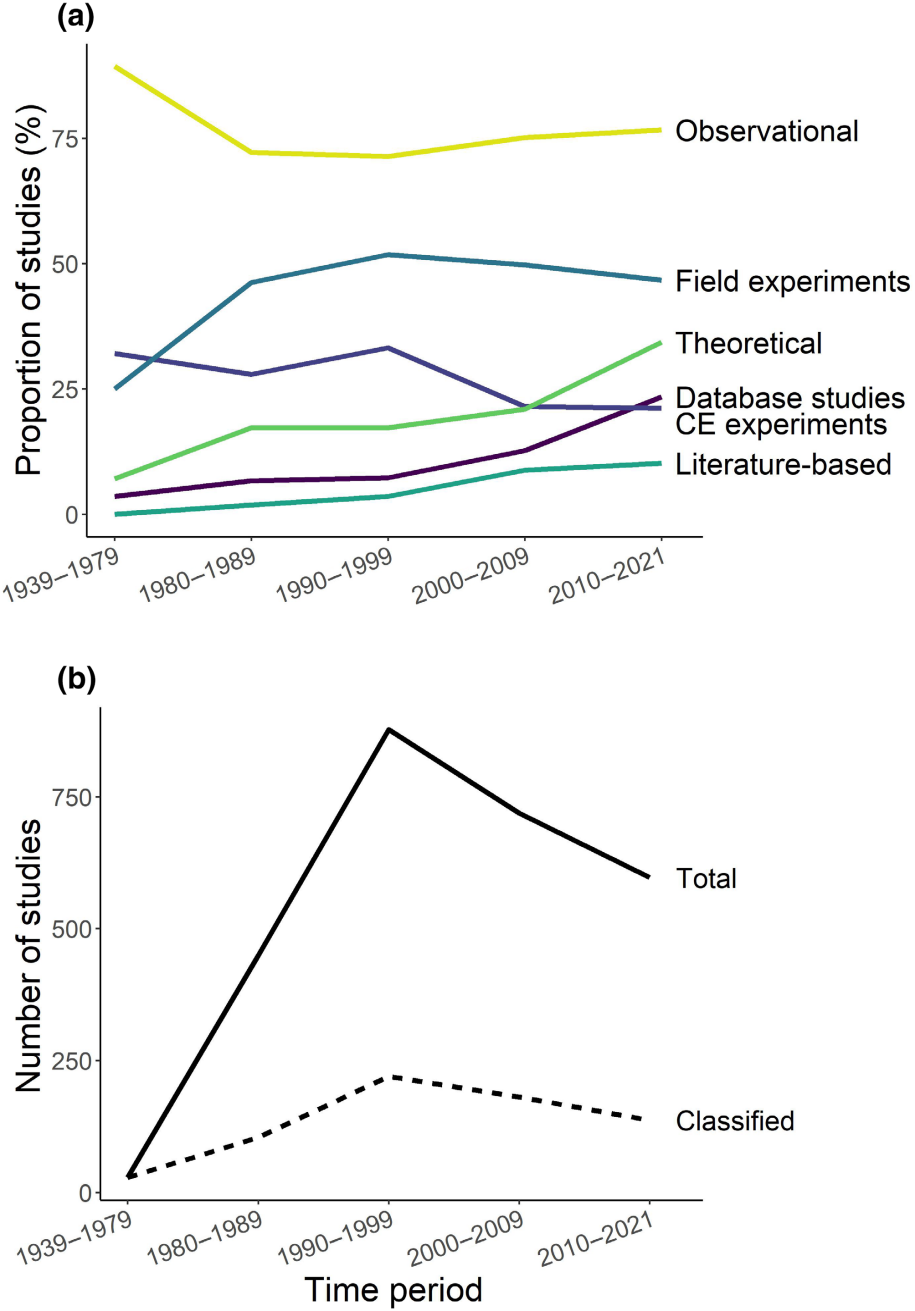
Trends in methodological approaches over time. (a) Proportion of studies (%) belonging to each methodology category in each decade. Note that totals may be >100%, as each dissertation could include portions of work in more than one category. (b) Total number of studies found (solid) and number classified (dashed) from each time period. CE experiments = controlled‐environment experiments.

Observational study was the most common research methodology overall, with the prevalence of this category remaining above 70% in all decades (Figure 1a, Table S1). The second and third most common categories were field experiments and controlled‐environment experiments, which were found in 48.2% and 26.7% of the dissertations, respectively. The remaining four categories, combined, were found in fewer than 40% of the studies. (Note that totals may be >100% since each dissertation could include portions of the work in more than one category.)

Over time, there was a slight decrease in the prevalence of controlled‐environment experiments, while literature‐based studies, database analyses, and theoretical studies increased substantially (Figure 1). Database analyses showed the most dramatic increase, going from 6.7% prevalence in the 1980s to 23.4% in the 2010s. Theoretical studies also increased noticeably, from 17.3% in the 1980s to 34.3% in the most recent decade. However, these increases were not accompanied by corresponding declines in other categories.

The total number of dissertations found increased up to the 1990s, then declined (Figure 1b, Table S1). All of the categories evaluated declined in total numbers, except for theoretical and database studies, which increased in total numbers as well as proportionately; literature‐based studies remained relatively constant over time.

### 3.2 The number of approaches used to collect data has increased

**FIGURE 2:**
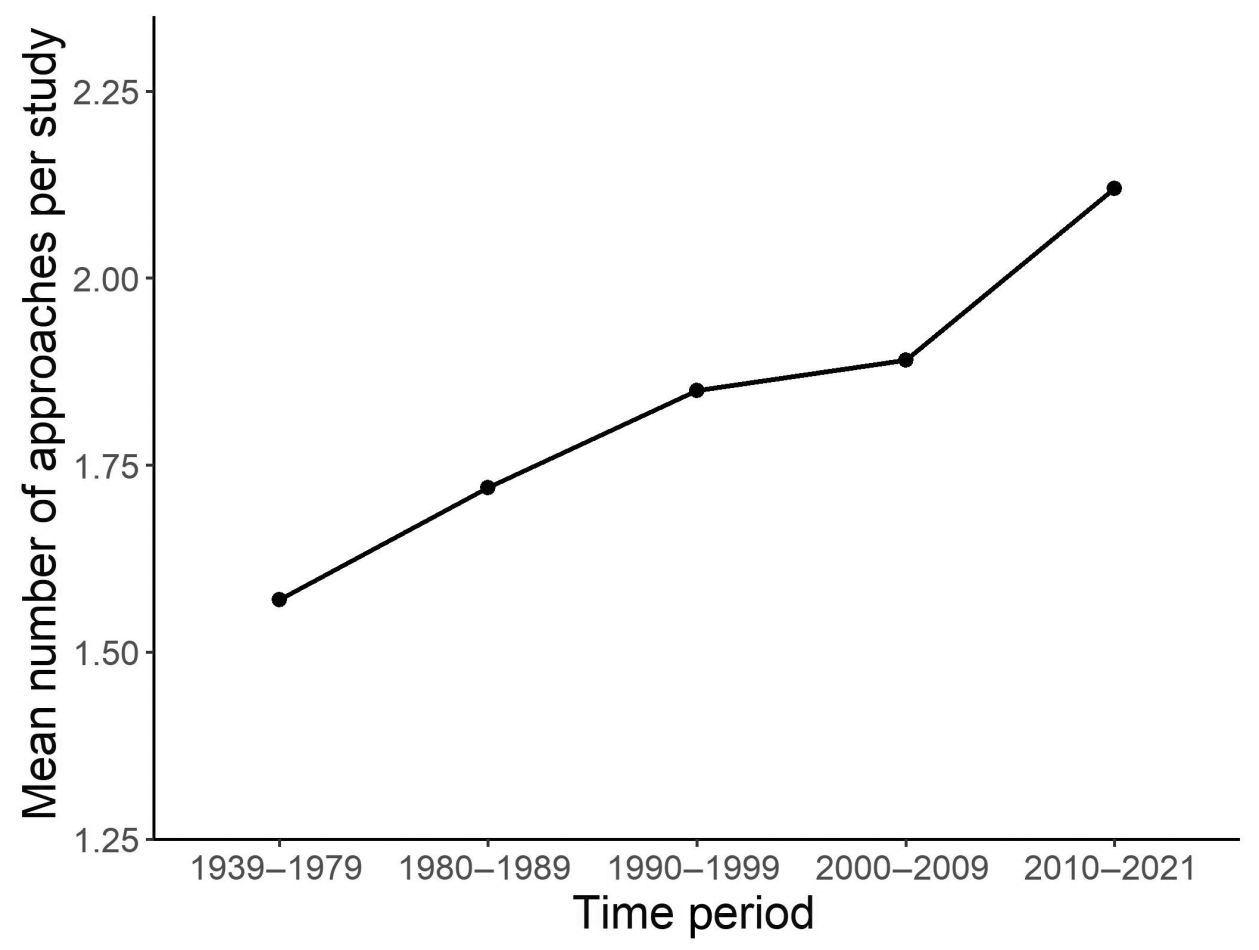
Mean number of approaches per study during each time period.

Across all years, most dissertations used 1–2 methodological approaches. The number of approaches per dissertation increased with time (Figure 2, Table S1). Dissertations from the 1980s had an average of 1.7 methodological categories per study, while by the 2010s, this had increased to 2.1 categories per study. Similarly, the proportion of studies with more than 2 approaches increased from 17% in the 1980s to 28% in the 2010s (Table S1).

### 3.3 Gender ratio in plant ecology has changed over time and differs with methodology

**FIGURE 3:**
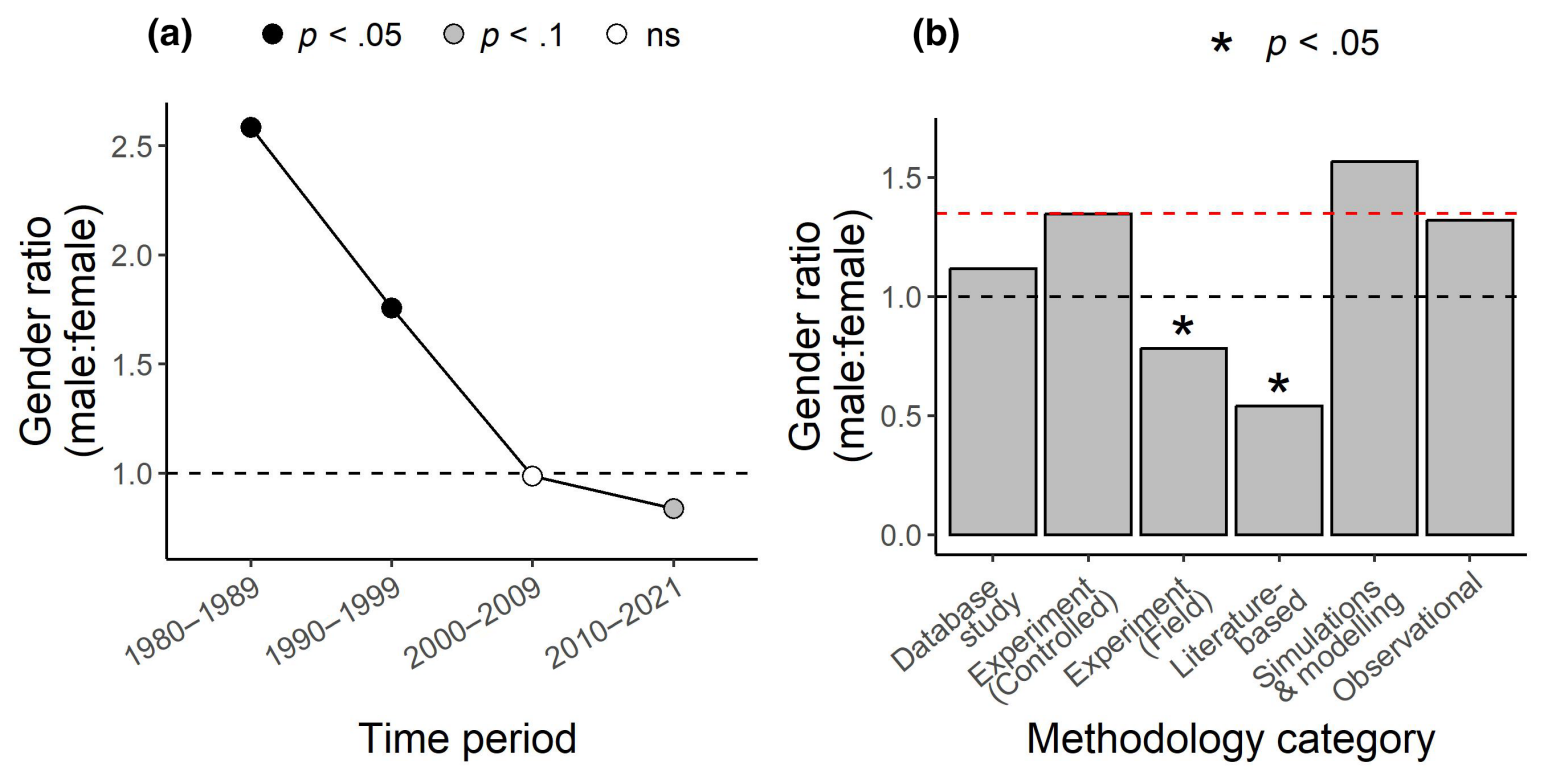
Gender ratio in plant ecology (a) over time and (b) across methodological categories. (a) Filled symbols indicate that the gender ratio was significantly different from a 1:1 ratio (*p* < .05), based on Chi‐squared goodness‐of‐fit tests. The black dashed line indicates a 1:1 ratio. (b) Stars (*) indicate whether the ratio was significantly different from the overall ratio of 1.35:1 male to female (*p* < .05), based on Chi‐squared goodness‐of‐fit tests. The black dashed line indicates a 1:1 ratio, and the red dashed line indicates the overall average ratio.

We were able to determine the gender of ~87% of the authors in our dataset. The overall gender ratio, across all years, was male‐biased, with 1.35 males for every female author. However, there was a large change over the decades (Figure 3a, Table 2). The gender ratio was strongly and significantly male‐biased in the 1980s and 1990s, but it became more or less equal in the 2000s and then slightly female‐biased in the 2010s.

**TABLE 2:** Gender ratio over time.

Decade	Number male	Number female	Ratio (male:female)	Chi‐squared statistic	*p*‐Value	Corrected *p*‐value
1980–1989	292	113	2.58:1	79.11	<2.2 × 10^−16^	**<8.8 × 10** ^**−16**^
1990–1999	490	279	1.76:1	57.89	2.77 × 10^−14^	**8.29 × 10** ^**−14**^
2000–2009	314	318	0.99:1	0.025	.87	.87
2010–2021	257	307	0.84:1	4.43	.035	.070

*Note*: Chi‐squared goodness‐of‐fit tests were used to determine whether the ratio significantly differed from 1:1 (equal male and female representation). *p*‐Values were corrected for multiple comparisons using the Holm–Bonferroni method. Bold indicates statistical significance (p<.05).

There were some differences in the gender ratios among methodological categories (Figure 3b, Table 3). Field experiments and literature‐based methods tended to be employed more by female authors, as the gender ratios in these methodology categories (0.8:1 and 0.5:1, respectively, favoring females) were significantly lower than the overall gender ratio. On the other hand, theoretical methods were used slightly more often by male authors than female authors (gender ratio of 1.6:1, favoring males), although this was not significantly different from the overall ratio (1.35:1).

**TABLE 3:** Gender ratio across different methodological categories.

Category	Number male	Number female	Ratio (male:female)	Chi‐squared statistic	*p*‐Value	Corrected *p*‐value
Observational	255	193	1.32:1	0.041	.84	>1
Controlled‐environment experiments	93	69	1.35:1	1.49 × 10^−5^	1.00	1.00
Field experiments	133	170	0.78:1	22.58	2.02 × 10^−6^	**1.21 × 10** ^**−5**^
Literature‐based	13	24	0.54:1	7.50	.0061	**.031**
Databases analyses	38	34	1.12:1	0.63	.43	>1
Theoretical (modeling & simulations)	83	53	1.57:1	0.74	.39	>1

*Note*: Chi‐squared goodness‐of‐fit tests were used to determine whether the ratio in a given category significantly differed from the overall gender ratio across all dissertations (1.35:1). *p*‐Values were corrected for multiple comparisons using the Holm–Bonferroni method. Bold indicates statistical significance (p<.05).

## 4 DISCUSSION

### 4.1 Approaches in plant ecology dissertations have retained their character and yet expanded

Examining the methodology used in plant ecology dissertations provides a unique perspective on change and stasis in the field over time. We found that observational studies and field experiments remain the two most commonly used approaches, even in the most recent decade. This contradicts the common belief that these traditional “boots‐on‐the‐ground” approaches are being replaced by big data studies. While there has been a large increase in database studies in recent years, this increase has not come at the cost of experimental and observational approaches.

Further, we found that dissertations in plant ecology became more multi‐faceted over time, with graduate students incorporating a greater variety of methodological approaches in their dissertations. We believe that this increase in the diversity of approaches is beneficial for the training of Ph.D. students and for the field as a whole. All approaches have strengths and drawbacks, such as trade‐offs between generality, precision, and realism. Therefore, using a variety of approaches to address the same question would provide more robust conclusions and greater insights than using a single approach alone. Moreover, graduate students are likely to learn a greater variety of skills from using multiple approaches, which may be beneficial for their future careers.

Some previous studies have similarly examined trends in methodological approaches in ecology or related fields (Asselin & Gagnon, 2015; Carmel et al., 2013; Geldmann et al., 2020; Lisón et al., 2020; Ríos‐Saldaña et al., 2018; Shorrocks, 1993; Spiegelberger et al., 2012), but none using doctoral dissertations. Almost all these studies analyzed journal articles instead of dissertations, except one that analyzed conference presentations (Geldmann et al., 2020). Only one of these studies, Spiegelberger et al. (2012), focused on the field of plant ecology specifically. Another focused on the broader field of ecology (Carmel et al., 2013). The rest examined slightly different fields, such as conservation science (Geldmann et al., 2020; Ríos‐Saldaña et al., 2018), or had a narrower scope, such as examining trends within a single journal (Asselin & Gagnon, 2015; Shorrocks, 1993) or within studies on a single taxa (Lisón et al., 2020). Nevertheless, they all found observational or field‐based methods to be the most dominant methodology. This consistency is rather remarkable, given the large variation in scope within this literature. However, some of these studies reported a lower prevalence of observational methods than what we found (Spiegelberger et al., 2012) and/or a decline in this category over time, with concurrent rises in newer approaches such as mathematical modeling, data analysis, and remote sensing (Geldmann et al., 2020; Ríos‐Saldaña et al., 2018; Shorrocks, 1993). This contradicts our findings, which show that the proportion of observational studies remained stable over time, even as other methodologies increased.

These differences between past studies and ours could be due to differences in scope and methodology. However, the temporal decline in observational and field‐based studies in the published literature may also indicate a rising publication bias against these approaches. Indeed, some of the above‐mentioned studies found that observational and fieldwork studies were less common in high impact factor journals as compared to lower impact factor journals (Carmel et al., 2013; Ríos‐Saldaña et al., 2018; Spiegelberger et al., 2012). This is concerning, as such a bias could hinder the dissemination of field‐based studies, which continue to be important for the progress of ecology (Sagarin & Pauchard, 2010).

### 4.2 The role of women in plant ecology has increased, slowly

Historically, women were largely excluded from science, technology, engineering, and mathematics (STEM) careers, and their lower participation in these fields remains a concern even today (Holman et al., 2018; UNESCO Institute for Statistics, 2020). However, in plant ecology, we found that currently, men and women are more or less equally represented at the doctorate dissertation level. This is in line with other studies on gender ratios in STEM higher education, particularly in the United States (Mann & DiPrete, 2013; National Science Board, NSF, 2022). For example, in the USA, women made up roughly 45% of the doctoral degree earners in STEM, and 52% in biological sciences in particular, in 2019 (National Science Board, NSF, 2022). But despite this, women represent only about 30% of STEM researchers globally (UNESCO Institute for Statistics, 2020). Similarly, only around 30% of STEM jobs in the United States are held by women (National Science Board, NSF, 2022). Women are also underrepresented among publication authors in botany (Holman et al., 2018) and distinguished conference speakers in ecology (Farr, 2017). Whether this inequality is due to a long career lag or to systemic factors that continue to block women's advancement is not clear.

There were both unexpected gender differences and a lack of differences between male and female doctoral students. We expected that theoretical approaches would be male‐biased, as mathematics and computation‐heavy STEM fields (such as physics, mathematics, and computer sciences) tend to be male‐dominated (National Science Board, NSF, 2022). However, we did not find a significant male bias in the use of this approach. On the other hand, literature‐based methods and field experiments were significantly more likely to be used by female researchers. It is not clear why this is the case. One possibility is that different research methods may be emphasized by Ph.D. advisors when training graduate students of different genders. Alternatively, male and female graduate students may simply prefer different methodologies. Further investigation is required to distinguish between these two possibilities. We were also unable to quantify how the gender ratios in each methodological category changed over time due to limited data.

### 4.3 Observational studies still dominate, but other approaches have nudged their way in

Despite recent concerns about the rise of database studies, our systematic review showed that observational studies and field experiments still dominate plant ecology. However, there has indeed been a rise in database studies, and this increase was particularly sharp between the 2000s and 2010s. Literature‐based studies and theoretical approaches have also increased. Overall, plant ecology dissertations have become more methodologically diverse, incorporating a larger variety of methodological approaches. This likely explains the increase in “newer” approaches without concurrent decreases in “traditional” approaches.

The gender ratio among plant ecology doctoral graduates has become more equal in recent decades, representing a great improvement since the 1980s. The gender ratio now appears to be trending toward female‐majority, mirroring the trends seen in other biological fields. Male and female doctoral students appear to have slightly different preferences for different methodological approaches, which should be investigated further. Comparisons of the results we found here to the published literature could be useful for identifying potential publication and gender biases in the published literature in plant ecology.

The decline in Ph.D. dissertations that we found after the 1990s in the field is concerning, but we do not know whether this represents a real trend or is a limitation of the database used. We are unable to check for the latter due to the lack of other large and easily accessible dissertation databases. Our study is also geographically biased, as most of the dissertations we found were from the United States and other Western, developed countries (Table S3). However, correcting for this and other potential biases in the ProQuest database would require extensive archival research and the inclusion of non‐English language dissertations, which is beyond the scope of this study.

Overall, our study provides insights into the way data are collected in plant ecology, particularly in early career stages, which can help track the fundamental basis for progress in ecology.

## AUTHOR CONTRIBUTIONS

**Urmi Poddar:** Data curation (lead); formal analysis (lead); investigation (lead); methodology (equal); supervision (supporting); visualization (lead); writing – original draft (equal); writing – review and editing (equal). **Kristi Lam:** Data curation (supporting); formal analysis (supporting); investigation (supporting). **Jessica Gurevitch:** Conceptualization (lead); funding acquisition (lead); methodology (equal); project administration (lead); resources (lead); supervision (lead); writing – original draft (equal); writing – review and editing (equal).

## CONFLICT OF INTEREST STATEMENT

The authors declare no competing interest.

## Supporting information


Data S1.

## Data Availability

The data and code used in this study are available on the Dryad Digital Repository (https://doi.org/10.5061/dryad.h44j0zprx).
