# Supplementary material for: Trends in research approaches and gender in plant ecology dissertations over four decades
Source: Ecol Evol. 2024 Jun 11;14(6):e11554. doi: 10.1002/ece3.11554 (PMC11165400; doi:10.1002/ece3.11554)
Supplement: Supplementary file 1 — Data S1. [file ECE3-14-e11554-s001.docx]

**Table S1:** Distribution of methodological approaches in plant ecology dissertations from each decade, calculated by taking a random sample of ~25% of relevant dissertations from each decade. Note that the same dissertation can fall into more than one category.  Obs - observational, ExpF – field experiments, ExpC – controlled experiments, Dat – database studies, Lit – literature-based studies, Mat – theoretical (mathematical modeling and simulations).

| **Time period** | **Total** | **Number Classified** | **Category (Count, Percentage)** | | | | | | **Mean no. of approaches per study** | **% studies with >2 approaches** |
| --- | --- | --- | --- | --- | --- | --- | --- | --- | --- | --- |
|  |  |  | **Obs** | **ExpC** | **ExpF** | **Lit** | **Dat** | **Mat** |  |  |
| 1939-1979 | 28 | 28 | 25,  89.3% | 9,  32.1% | 7,  25.0% | 0,  0.0% | 1,  3.6% | 2,  7.1% | 1.57 | 0% |
| 1980-1989 | 449 | 104 | 75,  72.1% | 29,  27.9% | 48,  46.2% | 2,  1.9% | 7,  6.7% | 18,  17.3% | 1.72 | 17% |
| 1990-1999 | 877 | 220 | 157,  71.4% | 73,  33.2% | 114,  51.8% | 8,  3.6% | 16,  7.3% | 38,  17.3% | 1.85 | 18% |
| 2000-2009 | 719 | 181 | 136,  75.1% | 39,  21.5% | 90,  49.7% | 16,  8.8% | 23,  12.7% | 38,  21.0% | 1.89 | 22% |
| 2010-2021 | 597 | 137 | 105,  76.6% | 29,  21.2% | 64,  46.7% | 14,  10.2% | 32,  23.4% | 47,  34.3% | 2.12 | 28% |
| Overall | 2670 | 670 | 498,  74.3% | 179,  26.7% | 323,  48.2% | 40,  6.0% | 79,  11.8% | 143,  21.3% | 1.88 | 20% |

**Table S2:** Comparing the effect of sample size on the observed distribution of methodological approaches in plant ecology dissertations from each decade. The proportion of dissertations in each category was calculated by first using ~20% of the relevant dissertations from each decade, and then adding an additional ~5% of the relevant dissertations from each decade. Observed proportions in each category with ~20% and ~25% of the dataset are shown below. Data from 1939-1979 are not shown as all dissertations from this period were classified in the first round. Obs - observational, ExpF – field experiments, ExpC – controlled experiments, Dat – database studies, Lit – literature-based studies, Mat – theoretical (mathematical modeling and simulations.

| **Time period** | **Number Classified** | | **Proportion in each category (%)**  **with 20% of dataset** | | | | | | **Proportion in each category (%)**  **with 25% of dataset** | | | | | |
| --- | --- | --- | --- | --- | --- | --- | --- | --- | --- | --- | --- | --- | --- | --- |
|  | **20%** | **25%** | **Obs** | **ExpC** | **ExpF** | **Lit** | **Dat** | **Mat** | **Obs** | **ExpC** | **ExpF** | **Lit** | **Dat** | **Mat** |
| 1980s | 90 | 104 | 74.4 | 27.8 | 47.8 | 2.2 | 6.7 | 18.9 | 72.1 | 27.9 | 46.2 | 1.9 | 6.7 | 17.3 |
| 1990s | 175 | 220 | 73.7 | 34.3 | 53.7 | 2.9 | 6.9 | 16.6 | 71.4 | 33.2 | 51.8 | 3.6 | 7.3 | 17.3 |
| 2000s | 145 | 181 | 77.2 | 22.1 | 48.3 | 9.0 | 11.7 | 18.6 | 75.1 | 21.5 | 49.7 | 8.8 | 12.7 | 21.0 |
| 2010s | 119 | 137 | 77.3 | 16.8 | 45.4 | 9.2 | 21.8 | 35.3 | 76.6 | 21.2 | 46.7 | 10.2 | 23.4 | 34.3 |
| Overall | 557 | 670 | 76.3 | 26.2 | 48.1 | 5.6 | 11.1 | 21.0 | 74.3 | 26.7 | 48.2 | 6.0 | 11.8 | 21.3 |

**Table S3**: Geographic distribution of dissertations in our dataset.

| **Country** | **Count (among relevant dissertations)** | **Percent of relevant dissertations** | **Count**  **(among classified dissertations)** | **Percent of classified dissertations** |
| --- | --- | --- | --- | --- |
| United States | 1788 | 66.97% | 437 | 65.22% |
| England | 361 | 13.52% | 93 | 13.88% |
| Sweden | 100 | 3.75% | 24 | 3.58% |
| Scotland | 93 | 3.48% | 28 | 4.17% |
| Canada | 81 | 3.03% | 24 | 3.58% |
| Ireland | 33 | 1.24% | 8 | 1.19% |
| Wales | 29 | 1.09% | 9 | 1.34% |
| Finland | 26 | 0.97% | 8 | 1.19% |
| Northern Ireland | 18 | 0.67% | 3 | 0.45% |
| Switzerland | 12 | 0.45% | 4 | 0.60% |
| Australia | 11 | 0.41% | 3 | 0.45% |
| South Africa | 10 | 0.37% | 0 | 0% |
| Other | 25 | 0.94%. | 8 | 1.19% |
| Unknown | 83 | 3.11% | 21 | 3.13% |
| **Total** | **2670** | | **670** | |
